# Independent attenuation correction of whole body [^18^F]FDG-PET using a deep learning approach with Generative Adversarial Networks

**DOI:** 10.1186/s13550-020-00644-y

**Published:** 2020-05-24

**Authors:** Karim Armanious, Tobias Hepp, Thomas Küstner, Helmut Dittmann, Konstantin Nikolaou, Christian La Fougère, Bin Yang, Sergios Gatidis

**Affiliations:** 1 Department of Radiology, Diagnostic and Interventional Radiology, University Hospital Tübingen, Hoppe-Seyler-Str. 3, 72076 Tübingen, Germany; 2 Institute of Signal Processing and System Theory, University of Stuttgart, Stuttgart, Germany; 3 Max Planck Institute for Intelligent Systems, Tübingen, Germany; 4 School of Biomedical Engineering & Imaging Sciences, King’s College London, St. Thomas’ Hospital, London, UK; 5 Cluster of Excellence iFIT (EXC 2180) “Image Guided and Functionally Instructed Tumor Therapies”, University of Tübingen, Tübingen, Germany; 6 Department of Radiology, Nuclear Medicine and Clinical Molecular Imaging, University Hospital Tübingen, Tübingen, Germany

**Keywords:** PET, Attenuation correction, Deep learning, Whole body

## Abstract

**Background:**

Attenuation correction (AC) of PET data is usually performed using a second imaging for the generation of attenuation maps. In certain situations however—when CT- or MR-derived attenuation maps are corrupted or CT acquisition solely for the purpose of AC shall be avoided—it would be of value to have the possibility of obtaining attenuation maps only based on PET information. The purpose of this study was to thus develop, implement, and evaluate a deep learning-based method for whole body [^18^F]FDG-PET AC which is independent of other imaging modalities for acquiring the attenuation map.

**Methods:**

The proposed method is investigated on whole body [^18^F]FDG-PET data using a Generative Adversarial Networks (GAN) deep learning framework. It is trained to generate pseudo CT images (CT_GAN_) based on paired training data of non-attenuation corrected PET data (PET_NAC_) and corresponding CT data. Generated pseudo CTs are then used for subsequent PET AC. One hundred data sets of whole body PET_NAC_ and corresponding CT were used for training. Twenty-five PET/CT examinations were used as test data sets (not included in training). On these test data sets, AC of PET was performed using the acquired CT as well as CT_GAN_ resulting in the corresponding PET data sets PET_AC_ and PET_GAN_. CT_GAN_ and PET_GAN_ were evaluated qualitatively by visual inspection and by visual analysis of color-coded difference maps. Quantitative analysis was performed by comparison of organ and lesion SUVs between PET_AC_ and PET_GAN_.

**Results:**

Qualitative analysis revealed no major SUV deviations on PET_GAN_ for most anatomic regions; visually detectable deviations were mainly observed along the diaphragm and the lung border. Quantitative analysis revealed mean percent deviations of SUVs on PET_GAN_ of − 0.8 ± 8.6% over all organs (range [− 30.7%, + 27.1%]). Mean lesion SUVs showed a mean deviation of 0.9 ± 9.2% (range [− 19.6%, + 29.2%]).

**Conclusion:**

Independent AC of whole body [^18^F]FDG-PET is feasible using the proposed deep learning approach yielding satisfactory PET quantification accuracy. Further clinical validation is necessary prior to implementation in clinical routine applications.

## Background

The introduction of combined PET/CT has not only resulted in increased diagnostic accuracy and simplified clinical work flows but importantly also led to a significant reduction in PET examination times using CT-based attenuation correction (AC) [1, 2]. In contrast to standalone PET scanners, which rely on time-consuming transmission scans for estimation tissue attenuation coefficients, rapidly acquired CT data can be used for this purpose in combined PET/CT [2]. Numerous studies have established and confirmed the quantitative accuracy of CT-based PET AC compared to the reference standard of transmission scan-based PET AC [3–5]. With the introduction of integrated PET/MR scanners, MR-based PET AC has been established as a further viable alternative providing satisfactory PET quantification accuracy [6]. To this end, a number of methods have been proposed including segmentation-based PET AC [6], atlas-based PET AC [7], and recently deep learning-based PET AC [8, 9], in all cases using anatomic MR information for estimation of tissue attenuation coefficients.

In certain situations, however, CT- or MR-based PET AC can be hampered by disturbing factors such as misregistration between PET and anatomic imaging (e.g., caused by patient displacement) and modality-specific artifacts including motion or metal artifacts [10–12]. In addition, specifically with respect to MR-based PET AC, MR image post processing for the purpose of PET AC may fail or generate secondary artifacts [11]. In these situations, artifacts occur on reconstructed PET images that can lead to inaccurate PET quantification and may even disturb clinical interpretation of image data. Furthermore, in certain diagnostic situations or research settings, it is conceivable that PET examinations are acquired without the necessity of additional CT imaging, e.g., when performing repetitive PET scans for dosimetry or when examining volunteers or children; in these cases, radiation exposure could be reduced by omitting CT scans.

In these situations—when CT- or MR-based AC is corrupted by artifacts or additional CT can be avoided—independent PET AC which does not require any other imaging modality for acquisition of the AC map and is thus only based on PET data would be a viable alternative. A number of methods have been proposed in this context. On the reconstruction side, the use of time-of-flight information has been shown to enable combined reconstruction and attenuation correction of PET data [13]. Due to the limited resolution and susceptibility for artifacts of the resulting attenuation maps, however, this approach has mainly be used for brain data or for improvement of CT- or MR-based AC rather than independent PET AC. Recently, deep learning-based estimation of attenuation maps from non-attenuation corrected PET (PET_NAC_) data has been proposed and demonstrated on brain PET data [14–16]. These methods use paired training data of PET_NAC_ and corresponding CT images to train a deep learning model to generate pseudo CTs from PET_NAC_ that are used for subsequent PET AC. For brain PET data, these methods have been shown to provide satisfactory quantitative results. Independent PET AC on whole body data, however, is significantly more challenging mainly due to considerably higher anatomical variability. Furthermore, artifacts of acquired AC maps occur much more frequently on whole body data.

The purpose of this study was thus to develop, implement, and evaluate a method for independent PET AC on whole body [^18^F]FDG-PET using a deep learning approach based on Generative Adversarial Networks (GANs).

## Methods

### Data

A total number of 125 whole body [^18^F]FDG-PET/CT scans acquired in 2018 and 2019 were retrospectively included in this study. Patient consent was waived by the institutional review board due to the retrospective and anonymized nature of data analysis. The 100 chronologically first PET/CT data sets were used as training data sets. The most recent 25 data sets with available PET sinogram raw data were used as test data sets. Patient information is summarized in Table 1.

**Table 1:** Patient characteristics (FUO, fever of unknown origin; HNSCC, head and neck squamous cell cancer; CUP, cancer of unknown primary site; CRC, colorectal cancer)

	Training cohort (*n* = 100)	Test cohort (*n* = 25)
**Age [years]**	62.5 ± 14	64 ± 13.3
**Gender**	Female 40, male 60	Female 12, male 13
**Weight [kg]**	75.1 ± 17.6	73.7 ± 16
**Height [m]**	1.7 ± 0.14	1.7 ± 0.12
**Diagnosis**	Lung cancer (27), melanoma (22), lymphoma (19), FUO (8), HNSCC (6), CUP (5), CRC (4), other (9)	Lung cancer (6), lymphoma (4), melanoma (3), CRC (3), CUP (2), esophageal cancer (2), cervical cancer (2), FUO (1), pancreatic cancer (1), ovarian cancer (1)

All data sets were acquired on a state-of-the-art clinical PET/CT scanner (Biograph mCT, Siemens Healthineers, Knoxville, Tennessee). Patients were positioned in prone position with arms elevated. In order to minimize involuntary motion, patients were embedded in a vacuum mattress. PET acquisition was performed 60 min after i.v.-injection of a body weight-adapted dose of 317.3 ± 8.6 MBq [^18^F]FDG.

In addition, a CT scan was acquired from the skull base to mid-thigh level for the purpose of attenuation correction. CT contrast agent was intravenously administered in all cases except for patients with contraindications.

All PET data sets were reconstructed using the following parameters: matrix size 400 × 400, 21 subsets and 2 iterations using a 2-mm Gaussian filter.

### Generation of pseudo CTs for independent AC

**Fig. 1:**
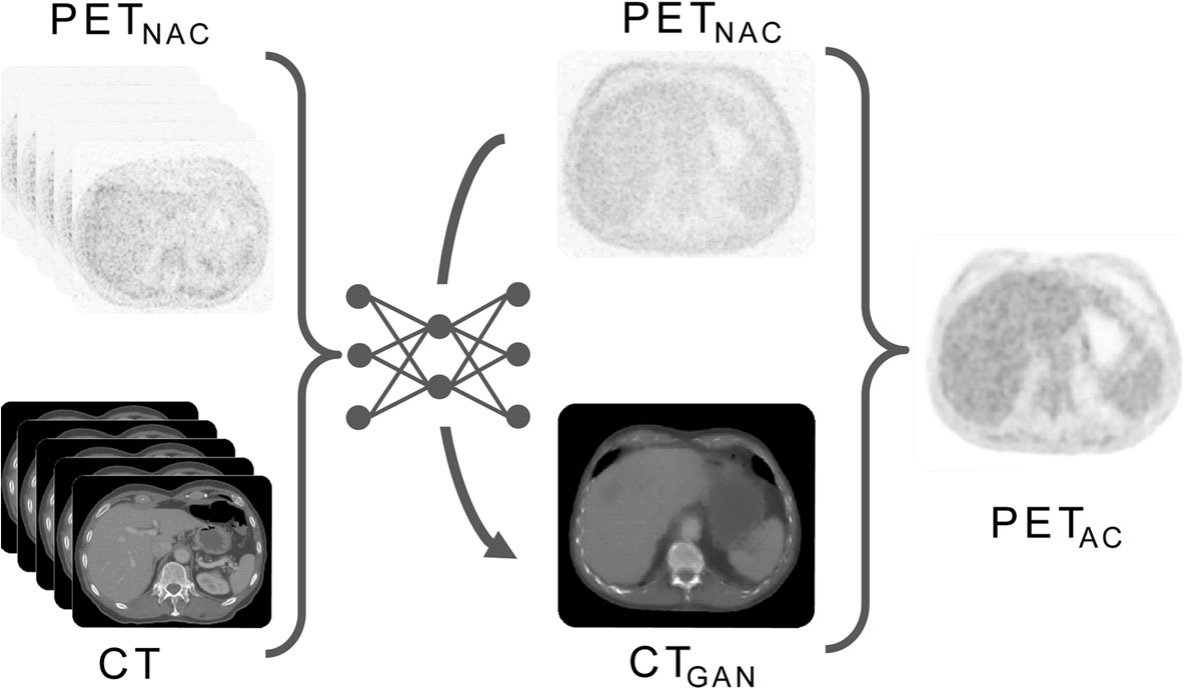
Process of independent whole body PET AC. Paired training data of non-attenuation corrected PET (PET_NAC_) and corresponding acquired CT are used to train a deep neural network to generate pseudo CT (CT_GAN_) from PET_NAC_. This pseudo CT can then be used for PET attenuation-correction resulting in an attenuation corrected PET data set (PET_AC_)

For the purpose of subsequent PET AC, a deep learning algorithm based on Generative Adversarial Networks (GANs) was implemented and trained to generate synthetic pseudo CT data (CT_GAN_) from non-attenuation-corrected PET data (PET_NAC_). In a second step, the generated synthetic CT_GAN_ images were used for PET AC (Fig. 1).

The basic architecture of the deep learning GAN framework was previously described in [14, 17]. It consists of two networks, a Generator network G and a Discriminator network D, trained simultaneously together in competition with each other. The Generator consists of four cascaded U-Net architectures trained in an end-to-end manner [18]. The Generator translates the input of 2D slices of PET_NAC_ (*y*_*NAC*_) to corresponding 2D slices of a synthetic CT image (${\hat{x}}_{CT}$) which is progressively refined via the cascaded encoder-decoder pairs of the U-Net architectures. On the other side, the Discriminator network functions as a binary classifier. It attempts to classify its inputs, the generated CT image (${\hat{x}}_{CT}$), and the corresponding ground-truth CT (*x*_*CT*_), as fake and real images, respectively. Both the Generator and Discriminator are trained in an adversarial manner end-to-end with the Generator attempting to improve the quality of the generated CTs in order to fool the Discriminator into making a wrong classification decision, whereas the Discriminator attempting to improve its classification performance. This adversarial training procedure is represented by the following min-max optimization task over the adversarial loss function:

$$ \underset{G}{\min \kern0.5em }\underset{D}{\max\ }{L}_{adv}=\mathrm{E}\left[\log D\left({x}_{CT},{y}_{NAC}\right)\right]+\mathrm{E}\left[\log \left(1-D\left({\hat{x}}_{CT},{y}_{NAC}\right)\right)\right] $$

To further enhance the quality of the resultant CT scans, additional non-adversarial loss functions are used to guide the Generator network to capture both the high and low-frequency components of the ground-truth CT scans. More specifically, the perceptual loss [19] and style-content losses [20] are used for training. The network was trained on a single Nvidia 1080ti GPU for approximately 80 h, while the inference time for each input PET_NAC_ was found to be approximately 100 ms.

### PET AC

PET data of the 25 test data sets were reconstructed twice each using the vendor-provided software: once using the corresponding acquired CT and once using the generated pseudo CT (CT_GAN_) resulting in two PET data sets per patient (PET_AC_ and PET_GAN_, respectively). In order to account for attenuation from scanner hardware and positioning devices and allow for a valid comparison, the background of CT was copied to CT_GAN_ prior to PET reconstruction.

### Data analysis

#### Qualitative analysis

CT_GAN_ and PET_GAN_ images of the test set were visually evaluated and compared to acquired CT and PET_AC_ respectively by a nuclear medicine physician (H.D.) and a radiologist (S.G.) in consensus. Visually detectable differences between CT_GAN_ and acquired CT as well as PET_GAN_ and PET_AC_ were recorded. In addition, voxel-wise difference maps (100∙(PET_GAN_ − PET_AC_)/PET_AC_) between PET_GAN_ and PET_AC_ were visually examined.

The occurrence and localization of areas of pathological focal [^18^F]FDG uptake related to primary tumors, metastases of inflammatory foci, was recorded on PET_GAN_ and PET_AC_ on the 20 test data sets.

#### Quantitative analysis

For quantitative evaluation, circular regions of interest (ROIs) were placed on PET images of the test set in the following anatomic structures using the Medical Imaging Interaction Toolkit version 2018.04 (MITK, German Cancer Research Center, Heidelberg, Germany): the left lung, right lung, mediastinal blood pool, liver, spleen, urinary bladder, 8th thoracic vertebral body (Th8), and 3rd lumbar vertebral body (L3). In addition, 50% isocontour volumes of interest (VOIs) were placed in areas of pathological focal PET uptake. All ROIs were defined on PET_AC_ and subsequently copied to PET_GAN_; 50% isocontours were adjusted after ROI transfer.

Mean Standardized Uptake Values (SUVs) were extracted from ROIs placed in the respective anatomic regions. Mean and maximum SUVs were extracted from VOIs in areas of pathological focal PET uptake. Deviations of SUVs in PET_GAN_ compared to PET_AC_ were quantified as difference (PET_GAN_ − PET_AC_), absolute difference (|PET_GAN_ − PET_AC_|), percent difference ($100\bullet \frac{\mathrm{PE}{\mathrm{T}}_{\mathrm{GAN}}-\mathrm{PE}{\mathrm{T}}_{\mathrm{AC}}}{\mathrm{PE}{\mathrm{T}}_{\mathrm{AC}}}$ ), and absolute percent difference ($100\bullet \frac{\left|\mathrm{PE}{\mathrm{T}}_{\mathrm{GAN}}-\mathrm{PE}{\mathrm{T}}_{\mathrm{AC}}\right|}{\mathrm{PE}{\mathrm{T}}_{\mathrm{AC}}}$).

In addition, in order to quantify differences in CT numbers between CT and CT_GAN_, organ ROIs were also transferred to CT and CT_GAN_ data sets, and mean Hounsfield units (HU) were extracted per ROI.

#### Statistical analysis

Numeric data are presented as mean ± standard deviation. SUV deviations of PET_GAN_ compared to PET_AC_ on the test set are graphically summarized as box plots showing the mean (circle), median (line), interquartile range (IQR, box), ± 1.5∙IQR (whiskers), and outliers (dots). Bland Altman analyses were performed comparing SUVs of areas of pathologic focal [^18^F]FDG uptake in PET_AC_ and PET_GAN_ on the test set as well as CT numbers between CT and CT_GAN_. Limits of agreement were calculated as the interval between ± 2 standard deviation.

## Results

### Qualitative analysis

**Fig. 2:**
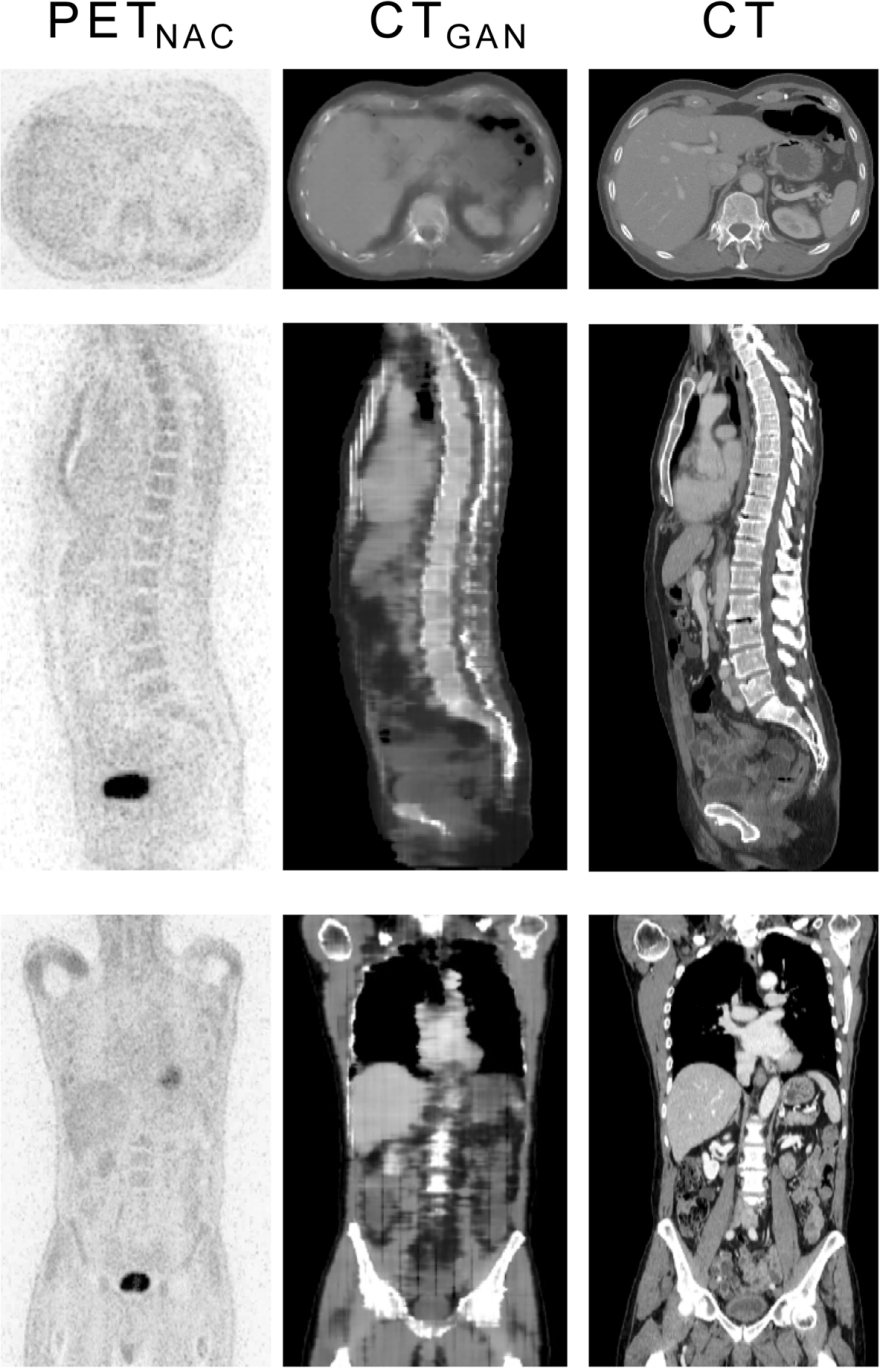
Representative data set showing non-attenuation-corrected [^18^F]FDG-PET (left), generated pseudo CT (middle), and acquired CT (right) in axial (top), sagittal (middle), and coronal (bottom) orientation. CT_GAN_ data—while capturing the coarse distribution of CT anatomy—showed blurring and step formation in *z*-direction on coronal and sagittal reconstruction as well as irregular depiction of anatomical details

In general, CT_GAN_ data showed a similar contrast and anatomy when visually compared to acquired CT data in all patients. The following artifacts were, however, detectable in all CT_GAN_ data sets (Fig. 2):

- Blurring and step formation in *z*-direction on coronal and sagittal reconstruction of CT_GAN_ due to 2D nature of image translation from PET_NAC_ to CT_GAN_. This was mostly evident along the spine as intervertebral disc space was not clearly identifiable.

- Irregular depiction of anatomic details such as the ribs, bowel configuration, and vessel structure.

- Deviating distribution air-filled intestine in CT_GAN_ compared to acquired CT.

A comparison of CT and CT_GAN_ of the remaining 24 test data sets is given in Supplement 1.

**Fig. 3:**
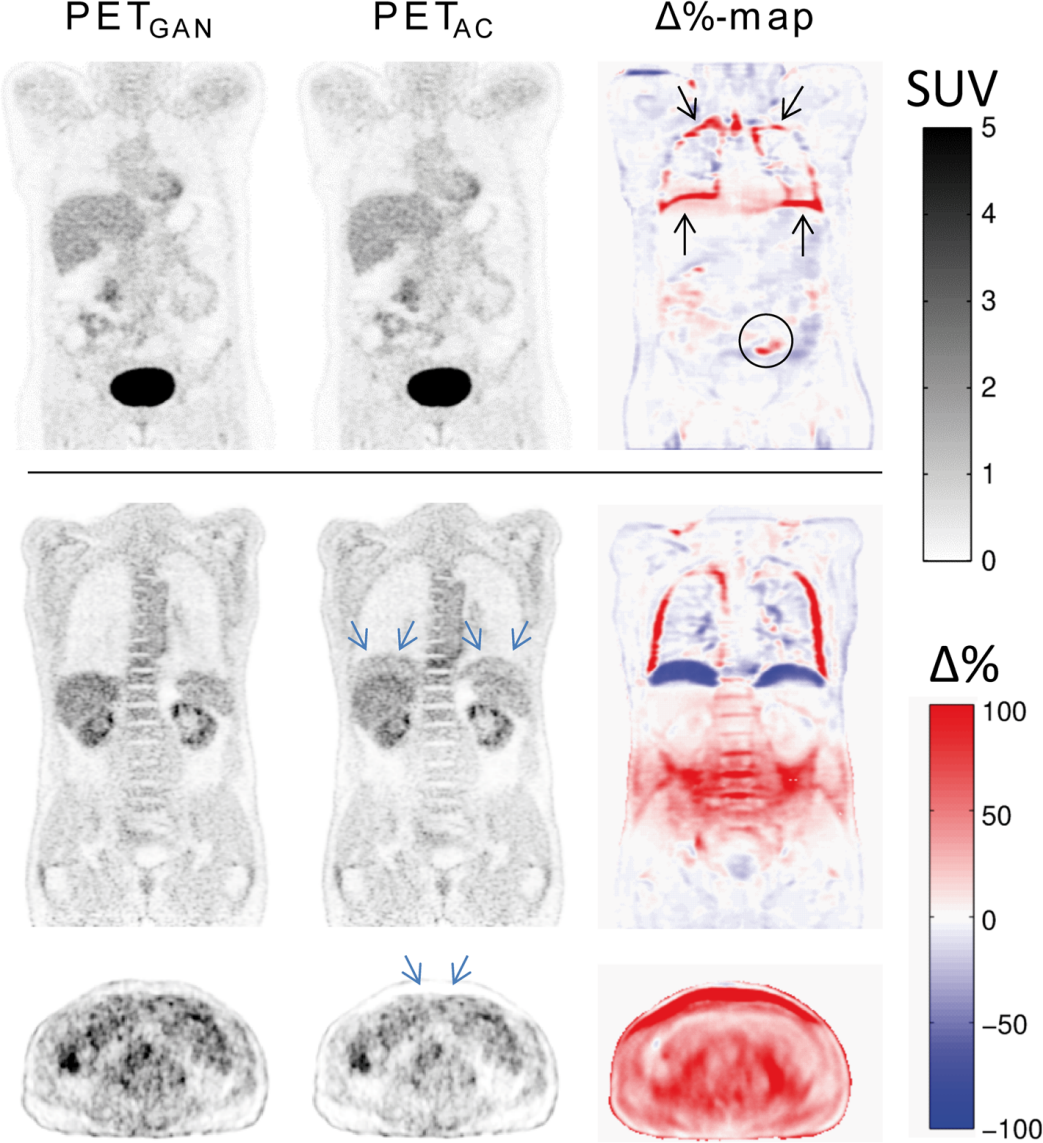
Two example data sets of PET data reconstructed with the generated pseudo CT (PET_GAN_, left), reconstructed using the acquired CT (PET_AC_, middle) and their voxel-wise percent difference map (Δ%-map, right). The top example shows typical results with no visually appreciable differences between PET_GAN_ and PET_AC_ in most anatomic regions and with more pronounced deviations localized along the lung border (black arrows) and in areas of air-filled bowel (black circle). The bottom example depicts a so-called banana artifact in PET_AC_ due to acquisition of PET and CT in different respiratory states resulting in relative overestimation of SUVs along the diaphragm and relative underesitmation of SUVs in the abdomen (blue arrows). These artifacts were not present on PET_GAN_

Visual comparison of PET_AC_ and PET_GAN_ revealed conceivable differences of varying degree especially along the lung borders at the diaphragm, the mediastinum, and the chest wall as well as in areas of air-filled intestine. In 3/25 data sets, significant differences were observed along the diaphragm with so-called banana artifacts in PET_AC_ that were not present in PET_GAN_ (Fig. 3). In 16/25 data sets, minor differences were observed along the lung borders of the diaphragm, the mediastinum, and the chest wall with sharper contours in PET_AC_ compared to PET_GAN_. In 13/25 data sets, minor differences in PET intensity distribution between PET_AC_ and PET_GAN_ were observed in areas of air-filled intestine.

Visual analysis of relative SUV deviation maps between PET_GAN_ and PET_AC_ revealed only minor SUV deviations of ± 5% in most anatomic areas. More pronounced relative deviations of varying degree were observed in 19/25 patients along the lung borders and in 13/25 patients in regions of air-filled intestine (Fig. 3) conforming the observations of visual comparison between PET_AC_ and PET_GAN_ described above.

In the three data sets with so-called banana artifacts, relative overestimation of SUV was observed on PET_AC_ along the diaphragm pointing to different respiratory states between PET (free breathing) and acquired CT (in these cases inspiration instead of desired expiration). In all three cases, these artifacts were not present in PET_GAN_. Furthermore, in these patients, difference maps revealed relative overestimation of SUVs in the abdominal region on PET_GAN_ compared to PET_AC_; again, this could be attributed to different respiratory states between PET (in expiration) and acquired CT (in inspiration) resulting in relatively reduced abdominal circumference on acquired CT images and thus underestimation of SUVs on PET_AC_.

Forty-one areas of pathologic focal [^18^F]FDG uptake were equally detected on PET_AC_ and PET_GAN_ in 15 patients in the following anatomic regions: the lung (9), mediastinal lymph nodes (8), bone (7), liver (5), abdominal lymph nodes (3), cervical lymph nodes (3), and other (6).

### Quantitative analysis

**Fig. 4:**
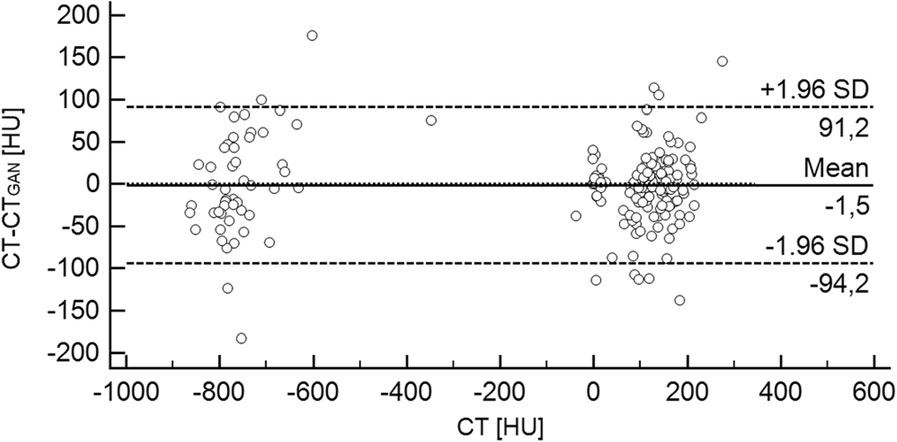
Bland Altman plot comparing mean Hounsfield units between CT and CT_GAN_ of all organ ROIs

Bland Altman analysis revealed a mean difference (± standard deviation) in CT numbers between CT and CT_GAN_ was − 1.5 ± 47.3 HU with limits of agreement between − 94.2 and 91.2 HU (Fig. 4). No systematic deviation of HU was observed for low or high density tissues.

**Fig. 5:**
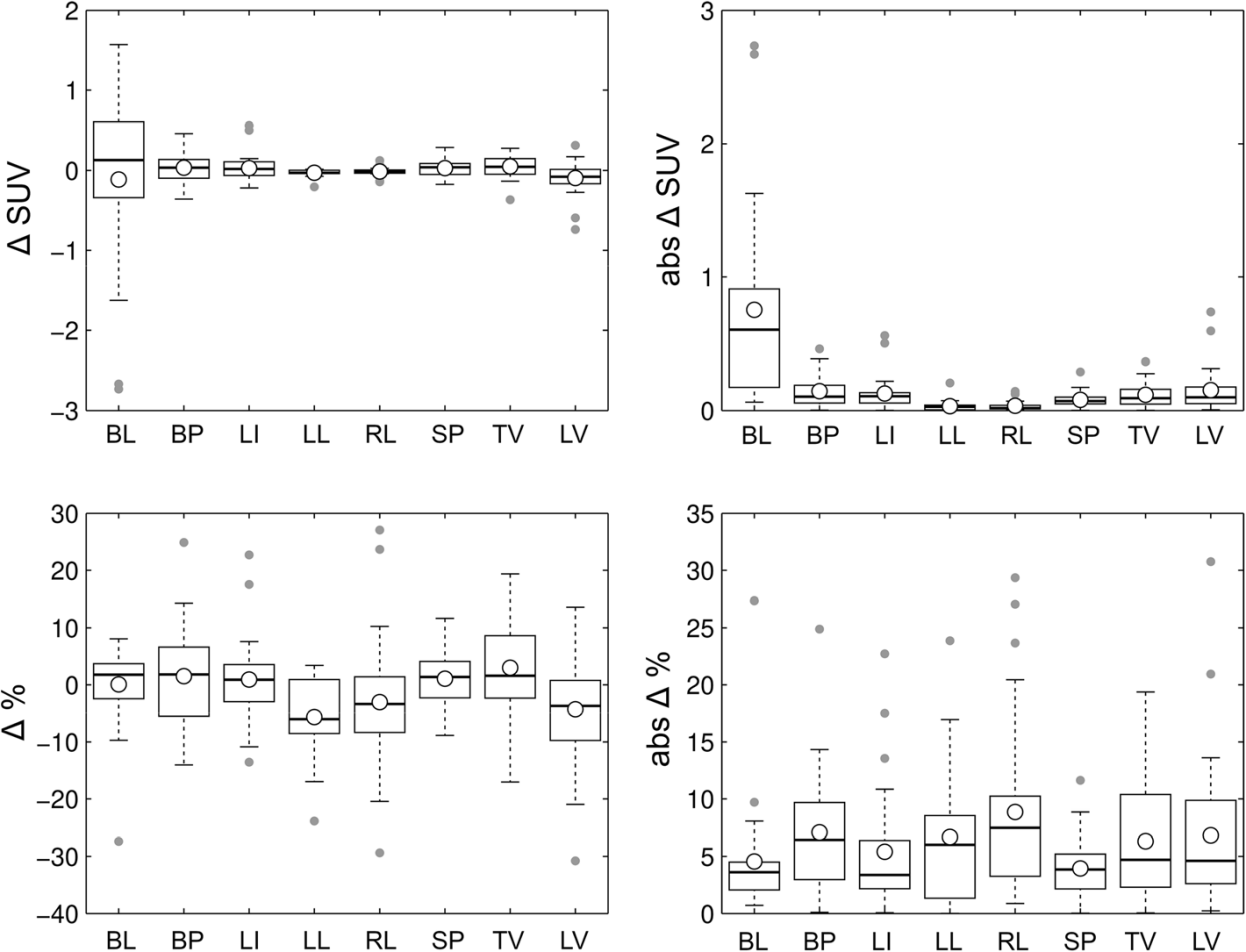
Deviation (upper left), absolute deviation (upper right), percent deviation (lower left), and absolute percent deviation (lower right) of SUVs in anatomic structures on PET_GAN_ compared to PET_AC_. (BL, urinary bladder; BP, blood pool; LI, liver; LL, left lung; RL, right lung; SP, spleen; TV, thoracic vertebra; LV, lumbar vertebra)

The mean deviation and mean percent deviation of SUVs over all anatomic structures and all data sets amounted to − 0.01 ± 0.4 and − 0.8 ± 8.6%, respectively. The highest mean SUV deviation was observed in the bladder (− 0.1 ± 1.1); the highest mean percent deviation was observed in the lungs (left lung − 5.6 ± 7.2%; right lung − 3 ± 11.7%). With respect to single data sets, percent SUV deviations ranged between − 30.7 (lumbar vertebra) and 27.1% (right lung). Results are summarized in Fig. 5.

Mean SUV_mean_/SUV_max_ deviation and mean percent SUV_mean_/SUV_max_ deviation of areas of pathologic focal [^18^F]FDG uptake amounted to 0.15 ± 0.8/0.13 ± 1.15 and 0.9 ± 9.2%/0.5 ± 9.2%, respectively. Deviation and percent deviations of SUV_mean_/SUV_max_ in individual data sets ranged between − 1.2/− 2.2 and 3.1/4.5 and between − 19.6%/− 18.6% and 29.2%/29.3%, respectively.

**Fig. 6:**
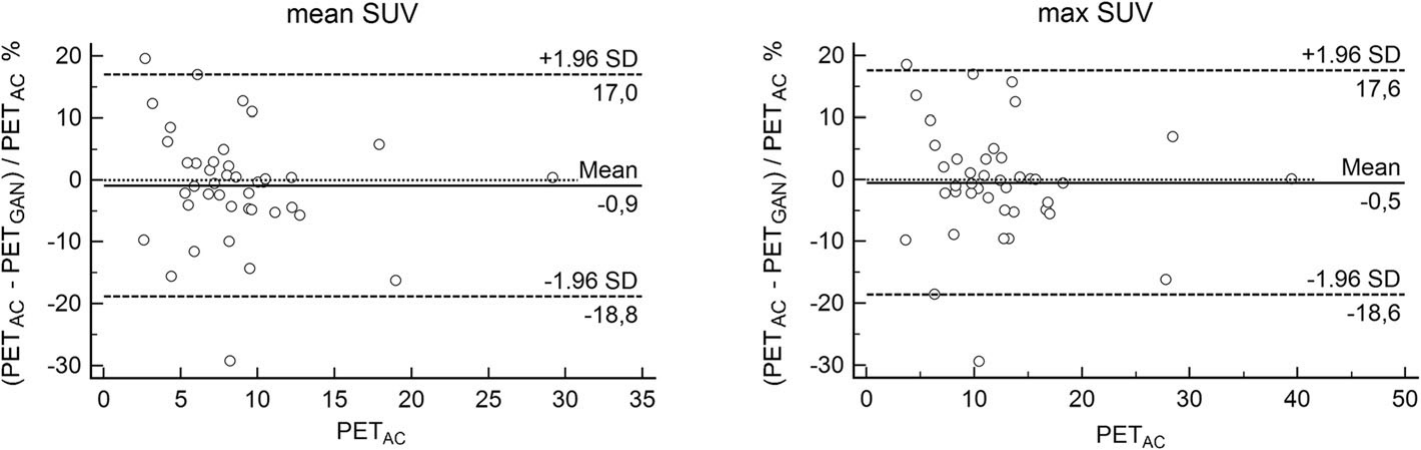
Bland Altman plots comparing mean (left) and maximum (right) SUVs of PET_AC_ and PET_GAN_ of 41 focal areas of pathologic tracer uptake

Bland Altman analysis revealed not systematic deviation of mean or maximum SUVs of areas of pathologic focal [^18^F]FDG uptake between PET_AC_ and PET_GAN_ (Fig. 6).

## Discussion

In this study, we implemented and evaluated a method for independent [^18^F]FDG-PET attenuation correction (AC) using a deep learning approach with Generative Adversarial Networks (GANs). We demonstrated the feasibility of this approach and observed satisfactory quantification results of PET SUVs in different anatomic regions and in pathologic lesions.

The quantitative results of our study are comparable to previous studies using deep learning methods for MR-based body PET AC [9, 21]. In contrast to these studies, we propose direct PET AC from non-attenuation-corrected PET data. Using the proposed method, we observed marked deviations of SUVs especially along the diaphragm and the lung border as well as in areas of air-filled bowel. Deviation along the lung and the diaphragm can be explained by different motion states of varying degree between PET and acquired CT; it can be assumed that independent PET attenuation correction is more reliable in these areas as it is solely based on PET data. Thus, the proposed approach excludes spatial displacement between PET data and the generated pseudo CT. Observed SUV deviations in the area of air-filled bowel segments can be explained by the difficulty of identifying air on PET data combined with the anatomical variability of the intestine leading to displacement on acquired CT.

In case gated PET data are acquired resulting in multiple PET images of different respiratory of cardiac states, PET-derived pseudo CTs could be computed separately for each of these states providing spatially aligned attenuation maps. As an extension of the presented method, such a framework could also be trained as a 4D model with 3 spatial dimension and one (gated) temporal dimension.

The PET quantification errors observed using independent PET AC are in the range of previously reported variation of SUVs caused by different PET reconstruction techniques [22] or observed using MR-based PET attenuation correction [6]. Importantly, PET quantification errors caused by MRI or CT artifacts have been reported as substantially higher [10, 23].

The presented method is limited to [^18^F]FDG-PET data as no other tracers were used in the training data set. An extension to non-[^18^F]FDG-data can be performed by retraining the GAN using respective paired training data sets. The results using non-[^18^F]FDG tracers can be expected to be in a similar range as long as sufficient anatomic information is present on PET data, which is to be expected for most clinical PET tracers. When highly targeting tracers are used however, results may potentially deteriorate.

Quantitative results of whole body independent PET AC may be further improved by using a 3D approach instead of slice-per-slice generation of pseudo CT as performed in this present study. The use of 3D models can be expected to provide more realistic pseudo-CT images especially perpendicular to the main imaging plane, which may result in more accurate estimation of attenuation coefficients. However, 3D approaches come with the limitation of substantially higher computational cost.

The proposed method can be applied as a back-up method on clinical PET/CT or PET/MR scanners in order to enable reliable PET AC also in cases of artifact-corrupted anatomic MR or CT images. A further potential application is the use in settings where only PET information is required, and radiation exposure from CT is unnecessary, e.g., in repeated scans, when examining volunteers or when examining children with already available anatomic imaging. To this end however, the proposed method requires further clinical validation also with respect to quantitative clinical scores such as the PERCIST or the Deauville-Score.

Despite the relatively high number of 100 training data sets, the variability of whole body PET/CT scans is likely not fully captured by the training data set. The addition of further cases, especially of abnormal anatomy and rare pathologies, will be necessary in order to increase model robustness. The validation of the proposed method in this study had a technical focus; thus, further clinical evaluation will be necessary.

## Conclusion

Independent AC of whole body [^18^F]FDG-PET is feasible using the proposed deep learning approach based on GANs. The proposed method achieved satisfactory PET quantification accuracy and does only depend on the acquired non-attenuation corrected PET data as input. Further clinical validation is necessary prior to implementation in clinical routine applications.

## Supplementary information

**Additional file 1:.** CTGAN compared to CT, data sets 1-6. CTGAN compared to CT, data sets 13-18. CTGAN compared to CT, data sets 19-24.

## Data Availability

The primary datasets analyzed during this study are not publicly available due to matters of data protection as these are clinical patient data. Upon reasonable request, secondary data of the performed analyses can be available from the corresponding author.

## Abbreviations

PET: Positron emission tomography
CT: Computed tomography
MR(I): Magnetic resonance (imaging)
AC: Attenuation correction
GAN: Generative Adversarial Networks
[^18^F]FDG: [^18^F]Fluorodeoxyglucose
SUV: Standardized Uptake Value
PET_NAC_: Non-attenuation-corrected PET
PET_AC_: Attenuation-corrected PET using CT AC
PET_GAN_: Attenuation-corrected PET using pseudo-CT AC
HU: Hounsfield unit
