# Supplementary material for: Independent attenuation correction of whole body [18F]FDG-PET using a deep learning approach with Generative Adversarial Networks
Source: EJNMMI Res. 2020 May 24;10:53. doi: 10.1186/s13550-020-00644-y (PMC7246235; doi:10.1186/s13550-020-00644-y)

Appendix: CT_{GAN} compared to CT, data sets 1-6

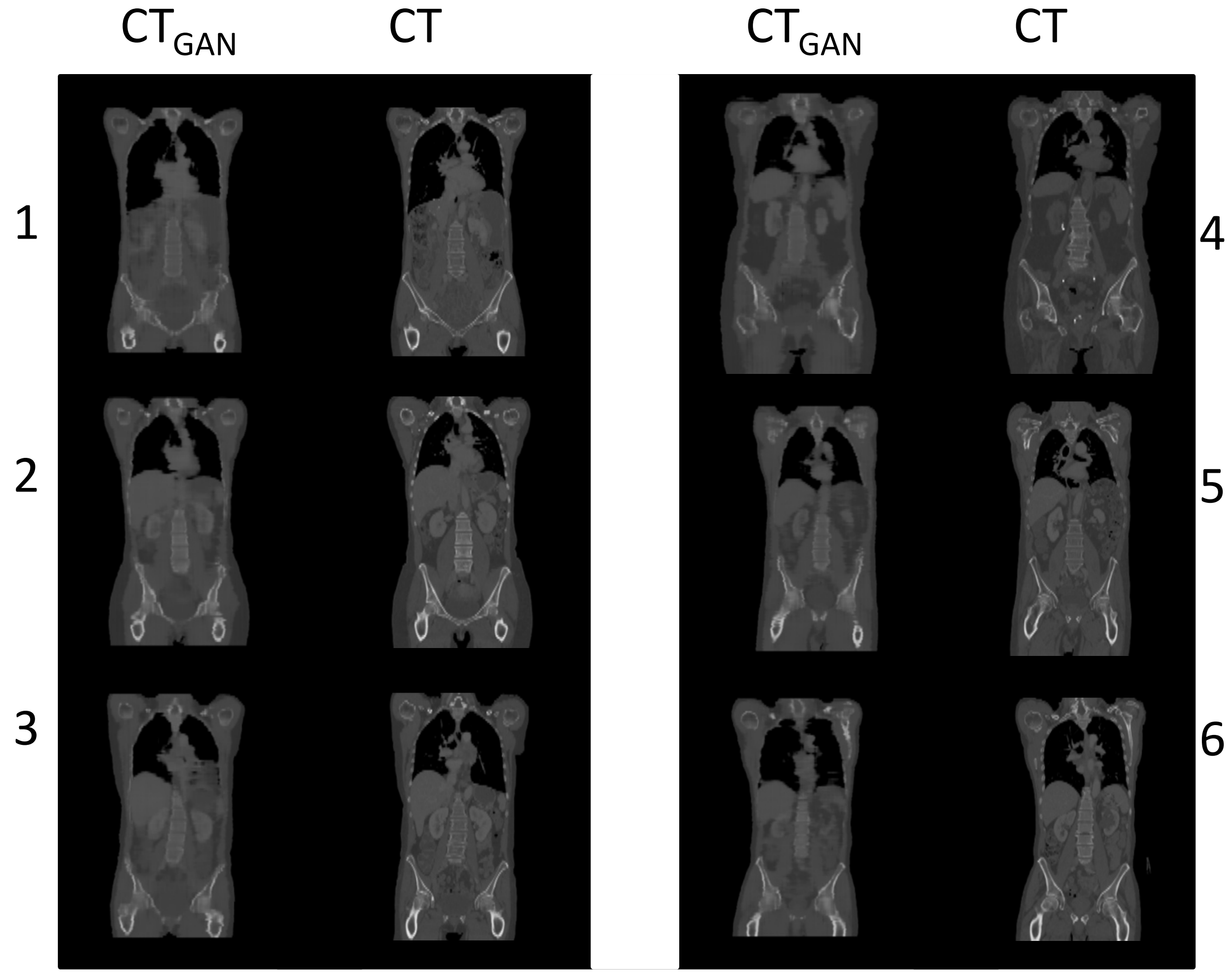

Appendix: CT_{GAN} compared to CT, data sets 13-18

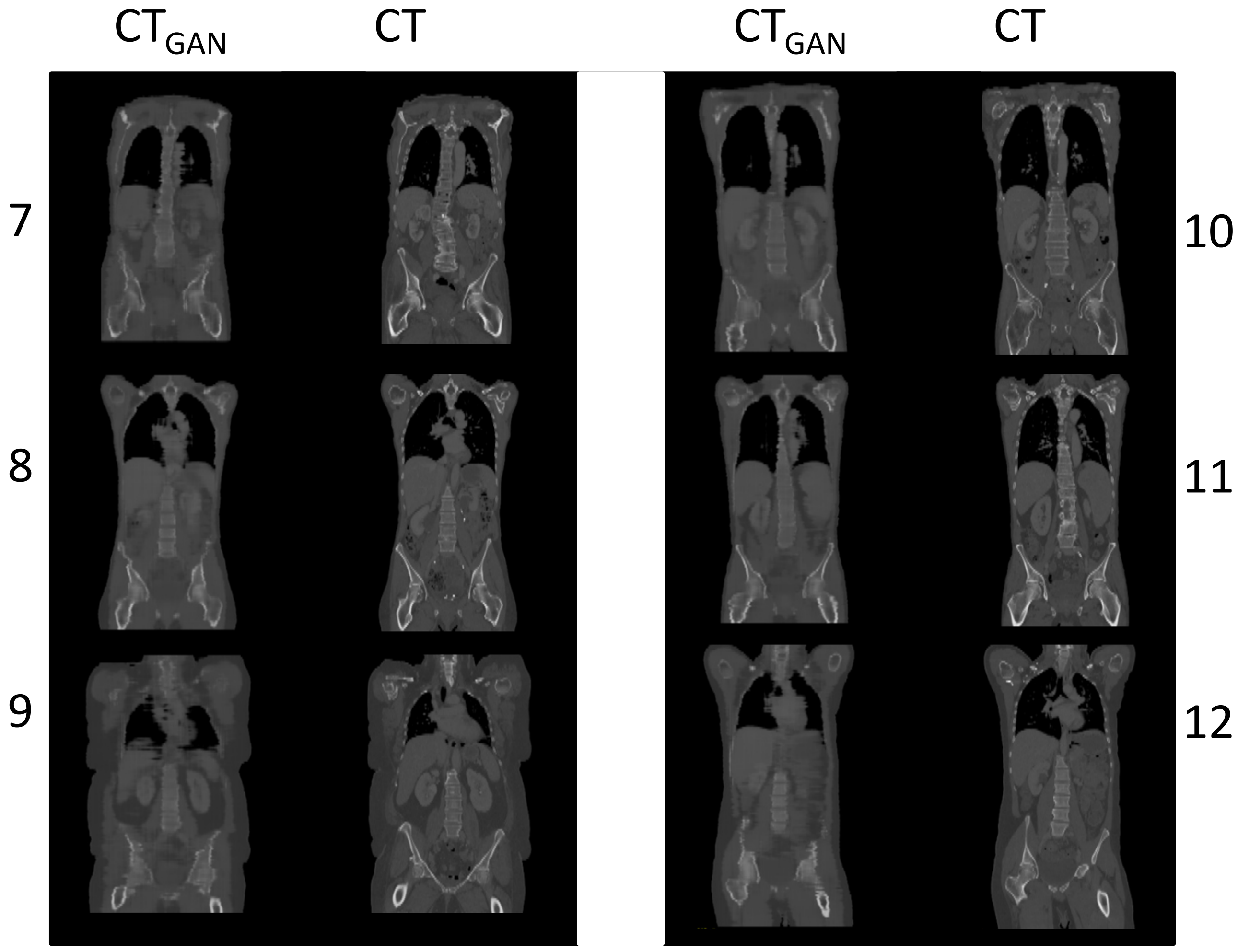

Appendix: CT_{GAN} compared to CT, data sets 13-18

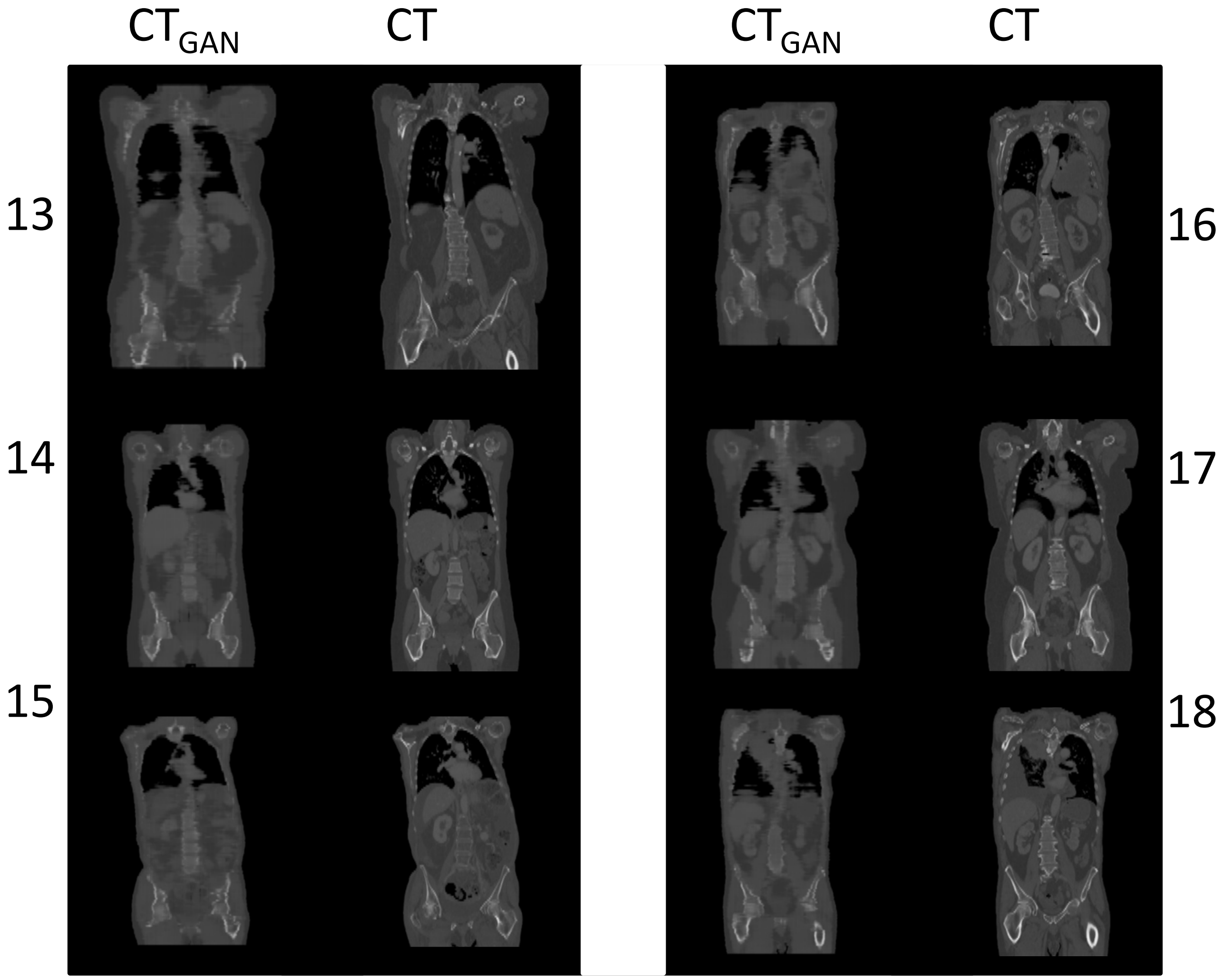

Appendix: CT_{GAN} compared to CT, data sets 19-24

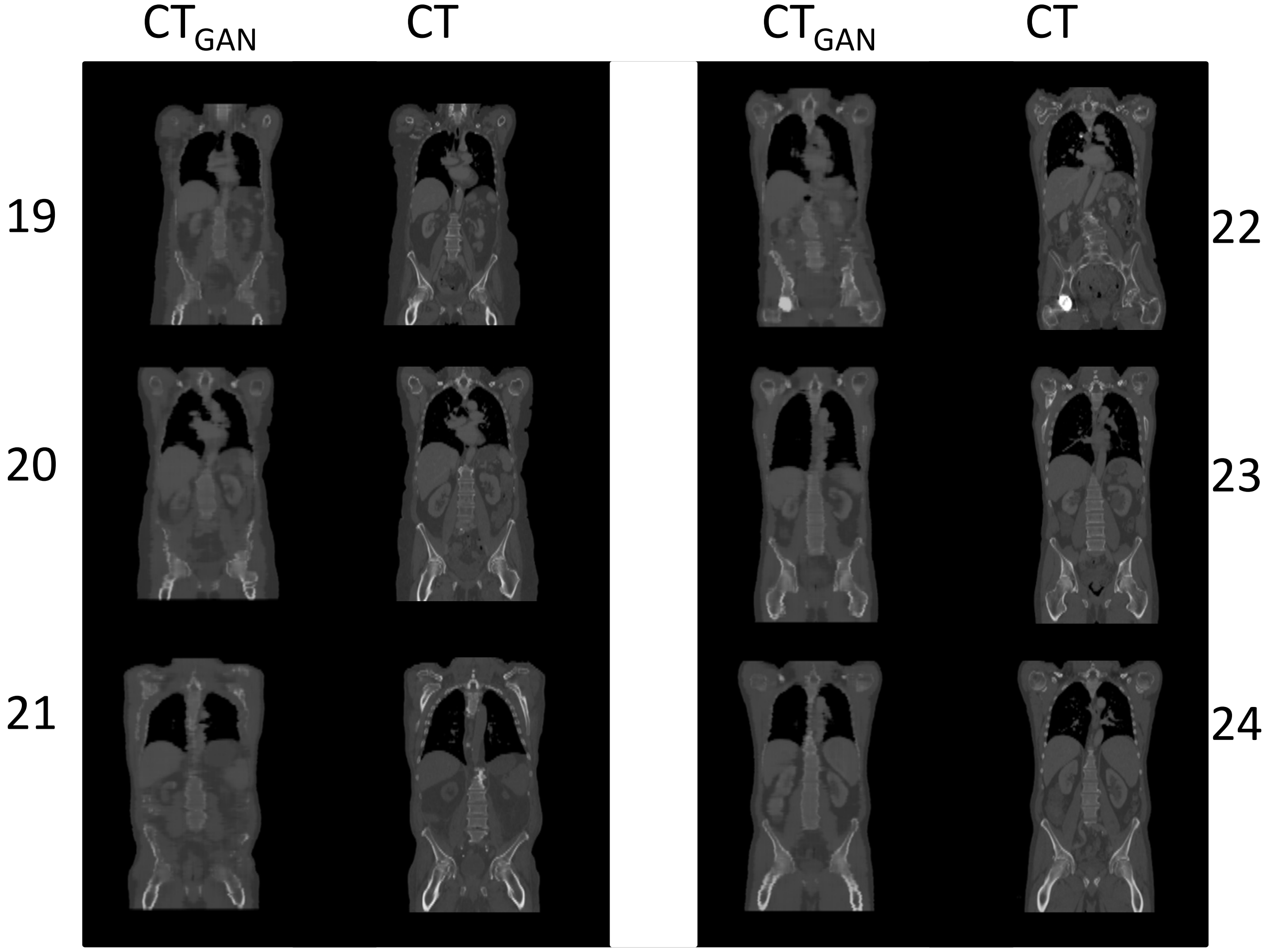

Supplement: Supplementary file 1 — Additional file 1:. CTGAN compared to CT, data sets 1-6. CTGAN compared to CT, data sets 13-18. CTGAN compared to CT, data sets 19-24. [file 13550_2020_644_MOESM1_ESM.pdf]
